# Accelerating Monte Carlo power studies through parametric power estimation

**DOI:** 10.1007/s10928-016-9468-y

**Published:** 2016-03-02

**Authors:** Sebastian Ueckert, Mats O. Karlsson, Andrew C. Hooker

**Affiliations:** Pharmacometrics Research Group, Department of Pharmaceutical Biosciences, Uppsala University, P.O. Box 591, 751 24 Uppsala, Sweden

**Keywords:** Non-linear mixed effect models, Hypothesis test, Power, Monte Carlo method, NONMEM

## Abstract

Estimating the power for a non-linear mixed-effects model-based analysis is challenging due to the lack of a closed form analytic expression. Often, computationally intensive Monte Carlo studies need to be employed to evaluate the power of a planned experiment. This is especially time consuming if full power versus sample size curves are to be obtained. A novel parametric power estimation (PPE) algorithm utilizing the theoretical distribution of the alternative hypothesis is presented in this work. The PPE algorithm estimates the unknown non-centrality parameter in the theoretical distribution from a limited number of Monte Carlo simulation and estimations. The estimated parameter linearly scales with study size allowing a quick generation of the full power versus study size curve. A comparison of the PPE with the classical, purely Monte Carlo-based power estimation (MCPE) algorithm for five diverse pharmacometric models showed an excellent agreement between both algorithms, with a low bias of less than 1.2 % and higher precision for the PPE. The power extrapolated from a specific study size was in a very good agreement with power curves obtained with the MCPE algorithm. PPE represents a promising approach to accelerate the power calculation for non-linear mixed effect models.

## Introduction

The calculation of the expected power of an experiment is a standard procedure often required by funding agencies, ethics boards or regulatory agencies. For simple statistical models, these calculations can be quickly performed using a simple analytic equation. For more complex models, analytic power calculations are often intractable and time consuming Monte Carlo methods need to be employed. This is especially true for non-linear mixed-effects models (NLMEM) which are frequently used within the paradigm of model-based drug development [7] due to their ability to handle the clustered, longitudinal nature of clinical trial data. In this work we present a new algorithm for power estimation which reduces computational effort considerably and evaluate its performance.

Power calculations for NLMEM are classically done by simulating a large number of datasets and re-estimating the simulated data with the planned analysis model to generate the distribution of the test statistic. This distribution is then used to obtain a power estimate. With this procedure, a large number of replicates is required for a stable estimate as each replicate contributes only dichotomous information (i.e., smaller or larger than the test threshold). This process is especially time-consuming if the procedure is to be repeated for different study sizes to obtain full power versus study size curves (power curves).

Existing alternatives to obtain power curves for NLMEM faster are Monte Carlo Mapped Power (MCMP) and Fisher information matrix-based power calculation (FIM-PC). MCMP, introduced by Vong et al. [14] and recently extended by Kloprogge et al. [6], uses the difference in the individual log-likelihood values derived from a large dataset simulated from a full model and subsequently re-estimated with the full and reduced models. The individual log-likelihood values are sampled and summed multiple times for each study size, and the power at a given study size is calculated as the fraction of individual log-likelihood sums larger than the critical value. FIM-PC for NLMEM was described by Retout et al. [11] and studied further by Ueckert et al. [13], it uses the theoretical relationship between the expected information matrix and the Wald test to compute the power curve.

The method presented in this work estimates an unknown parameter in the theoretical distribution of the test statistic under the alternative hypothesis and scales this estimate to obtain the power at different study sizes. Unlike MCMP, the algorithm does not require any special preparation of the dataset nor the calculation of the expected Fisher information matrix as FIM-PC. The algorithm will be referred to as parametric power estimation (PPE).

In this paper, we first introduce the PPE algorithm as well as a bootstrap procedure to evaluate uncertainty in the power estimate and a diagnostic to validate the underlying assumptions of the algorithm. Afterwards, we evaluate the proposed methods for a diverse set of NLMEM, for both continuous and discrete outcomes. The reference for our evaluation constitutes the classical, purely Monte Carlo-based way of estimating power, we will refer to this algorithm as Monte Carlo power estimation (MCPE). Finally, we demonstrate the practical use of our algorithm by applying it to a hypothetical disease progression example using the statistical software toolkit Perl speaks NONMEM (PsN).

## Methods

### Notation

#### Non-linear mixed effect models

Let $y_i$ be a vector of $n_i$ observations for individual *i* ($i=1,\ldots ,N$) and *y* be the vector of all observations ($y=(y_1,\ldots ,y_N)^T$). It will be assumed that observation *j* for individual *i* can be described through a NLMEM of the form

1 $$\begin{aligned} y_{ij} = f(t_{ij},\theta ,\eta_i,z_{ij}) + \varepsilon_{ij} \end{aligned}$$

when $y_{ij}$ is a continuous outcome or, in case $y_{ij}$ is discrete, through

2 $$\begin{aligned} P(y_{ij}|\eta_i) = h(t_{ij},\theta ,\eta_i,z_{ij}) \end{aligned}$$

where *f* and *h* are non-linear functions, $t_{ij}$ is the time of observation *j*, $\theta$ is a vector of fixed effect parameter, $\eta_i$ is a vector of subject-specific random effect parameter, $z_{ij}$ is a vector of covariates and $\varepsilon_{ij}$ is the residual error random effect. Both random effects are assumed to follow a normal distribution with mean 0 and covariance matrix $\Omega$ and $\Sigma$ for $\eta_i$ and $\varepsilon_{ij}$ respectively. Furthermore, let $\Theta =(\theta ,\Omega ,\Sigma )^T$ denote the vector of all unknown parameters.

#### Hypothesis testing and power

In the framework of NLMEM, a simple two-sided test for a fixed effect parameter $\theta_H$ can be formalized as

3 $$\begin{aligned} \text{H}_0: \theta_\text{H}=\theta_\text{H}^0 \nonumber \\ \text{H}_1: \theta_\text{H} \ne \theta_\text{H}^0 \end{aligned}$$

where $\text{H}_0$, $\text{H}_1$ are the null and alternative hypothesis and $\theta_\text{H}^0$ is the parameter value under the null hypothesis.

In the maximum likelihood (ML) framework, hypothesis tests are performed using a test statistic $t(\cdot )$ which depends on the ML estimate $\hat{\Theta }$. Two tests with different test statistics are considered in this work: the log-likelihood ratio (LLR) test and the Wald test. The LLR test evaluates the evidence for the null hypothesis in the log-likelihood domain using the test statistic

4 $$\begin{aligned} t_\text{LLR}\left( \hat{\Theta }\right) =\mathscr {L}\left( \hat{\Theta },y\right) -\mathscr {L}\left( \hat{\Theta }^0,y\right) \end{aligned}$$

where $\mathscr {L}(.)$ denotes the log-likelihood of the observed data *y* at the unrestricted maximum likelihood estimate $\hat{\Theta }=(\hat{\theta },\hat{\theta }_\text{H},\hat{\Omega },\hat{\Sigma })$ and the restricted maximum likelihood estimate $\hat{\Theta }^0=(\hat{\theta },\hat{\theta }^0_\text{H},\hat{\Omega },\hat{\Sigma })$, respectively. Commonly, the term full model is used to refer to the model estimated without restriction and the term reduced model to refer to the one estimated with the restriction $\theta_\text{H}=\theta_\text{H}^0$.

Rather then on the log-likelihood domain, the Wald test considers the evidence for the null hypothesis in the domain of the parameters using the formula

5 $$\begin{aligned} t_\text{Wald}\left( \hat{\Theta }\right) = \frac{\left( \hat{\theta }_\text{H}-\theta_\text{H}^0\right) ^2}{\mathrm {Var}\left( \hat{\theta }_\text{H}\right) } \end{aligned}$$

where $\mathrm {Var}( \hat{\theta }_\text{H})$ denotes the variance of $\hat{\theta }_\text{H}$ which is generally determined from the inverse of the observed Fisher information matrix $I^{-1}( \hat{\Theta })_{\text{H},\text{H}}$.

Both LLR and Wald test asymptotically follow a chi-square distribution with *k* degrees of freedom given that the null hypothesis is true [3]. Hence, both tests will reject the null hypothesis if $t( \hat{\Theta }) >\chi_{k,1-\alpha }^2$ where $\chi_{k,1-\alpha }^2$ is the $1-\alpha$ quantile of the chi-square distribution with *k* degrees of freedom. In this setting, the probability of correctly rejecting the null hypothesis given a specific alternative $\theta_\text{H}=\theta_\text{H}^*$ is called the power of the test $\pi$, i.e.

6 $$\begin{aligned} \pi = P\left( t\left( \hat{\Theta }\right) >\chi_{k,1-\alpha }^2|\theta_\text{H}=\theta_\text{H}^*\right) \end{aligned}$$

The power of a study is dependent on its design $\Xi$, where $\Xi$ is the set of all individual designs $\xi_i$, i.e. $\Xi =\{\xi_1,\ldots ,\xi_N\}$. In this work, mostly the influence of the number of subjects *N* on power will be studied and denoted $\pi (N)$.

### Monte Carlo power estimation

The MCPE algorithm estimates the power of a future trial by simulating $S_M$ datasets according to the planned study design, subsequently re-estimating the simulated datasets with the intended analysis model and finally calculating the test statistic for each replicate. The power estimate is then the fraction of times the null hypothesis was rejected. The LLR test is used more frequently for MCPE studies as it can be numerically challenging and more time consuming to obtain the observed Fisher information, required by the Wald test, for each of the replicates.

#### Power versus study size curves

Estimating the power for different study sizes *N* is a common task when planning a trial and can be accomplished by applying the MCPE algorithm for a predefined grid of study sizes $\{N_1,\ldots ,N_K\}$. The procedure for power estimation through the LLR test-based MCPE algorithm is described in Algorithm 1.
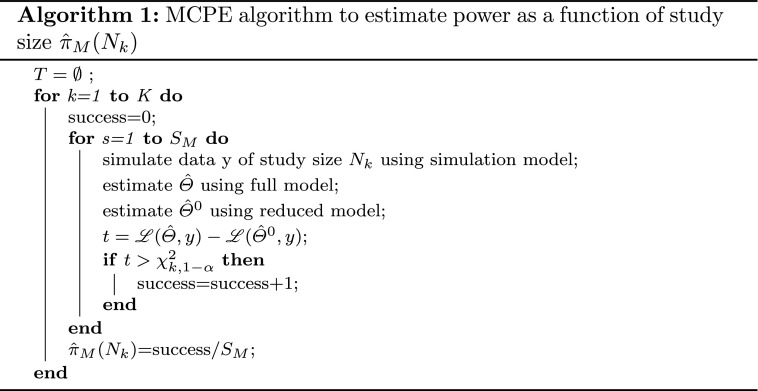


### Parametric power estimation algorithm

Under the alternative hypothesis $\text{H}_1$, LLR and Wald test statistic asymptotically follow a non-central chi-square distribution with *k* degrees of freedom and non-centrality parameter $\lambda$ given as

7 $$\begin{aligned} \lambda = \left( \theta_\text{H}-\theta_\text{H}^0\right) ^2\mathcal {I}^{-1}_{\text{H},\text{H}} \end{aligned}$$

where $\mathcal {I}^{-1}_{\text{H},\text{H}}$ is the entry for $\theta_\text{H}$ from the inverse of the expected Fisher information matrix [3].

The PPE algorithm estimates the unknown non-centrality parameter $\lambda$ from a sample of test statistics using maximum likelihood estimation. Let $f_{\chi ^2}(t,k,\lambda )$ denote the probability density function of the non-central chi-square distribution with *k* degrees of freedom and non-centrality parameter $\lambda$, and *T* a vector of LLR test statistics, then an estimate of the non-centrality parameter $\hat{\lambda }$ can be obtained via

8 $$\begin{aligned} \hat{\lambda }={arg\,max}_\lambda \sum_{s = 1}^{S_P} \log {f_{\chi ^2}(t,k,\lambda )} \end{aligned}$$

Based on $\hat{\lambda }$ the power is estimated as

9 $$\begin{aligned} {\hat{\pi}}_P=1-F_{\chi ^2}(\chi ^2_{1-\alpha , k},k,\hat{\lambda }) \end{aligned}$$

where $F_{\chi ^2}$ is the cumulative distribution function of the non-central ${\chi ^2}$ distribution and $\chi ^2_{1-\alpha , k}$ is the $1-\alpha$ quantile of the chi-square distribution.

#### Power versus study size curves

The expected information matrix for parameters $\Theta$ and population design $\Xi$ consisting of $N_k$ subjects with identical design variables $\xi$, is given as $N_k$ times the individual information matrix $\mathcal {I}(\Theta , \xi )$, i.e.

10 $$\begin{aligned} \mathcal {I}(\Theta , \Xi ) = N_k \mathcal {I}(\Theta , \xi ) \end{aligned}$$

For power curves, generally a reference design $\Xi_\text{ref}$ is postulated and replicated to arrive at different study sizes. Hence, combining Eqs. 10 and 7 yields an expression to scale the non-centrality parameter $\lambda_\text{ref}$ obtained for $N_\text{ref}$ subjects with population design $\Xi_\text{ref}$ to any study size $N_k$. The expression is given as

11 $$\begin{aligned} \lambda_k = \frac{N_k}{N_\text{ref}} \lambda_\text{ref} \end{aligned}$$

It should be noted that this equation does not require all subjects to have the same study design, but only assumes the reference design $\Xi_\text{ref}$ (potentially including different groups, etc.) to be replicated for different study sizes.

Combining Eq. 11 with the algorithm outline in the previous section yields the PPE algorithm for power curves which is presented in Algorithm 2.
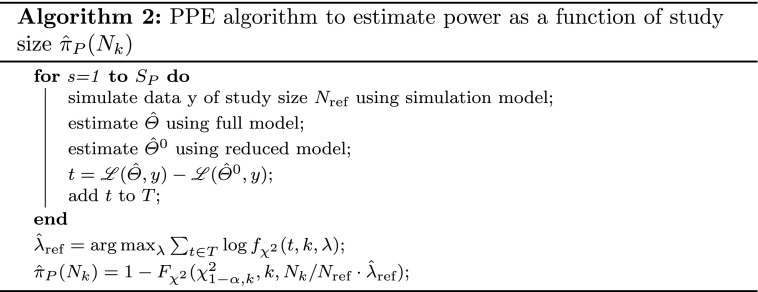


#### Bootstrap procedure to evaluate Monte Carlo uncertainty

The precision of the estimates from the PPE algorithm depend on the number of Monte Carlo samples $S_P$ used for the non-centrality parameter estimation. A practical way of evaluating this influence is through implementation of a parametric bootstrap procedure [2].

The bootstrap procedure first estimates $\lambda_\text{ref}$ as outlined in Algorithm 2. In the second step, *B* sets $T_b$ of random numbers, each of size $S_P$, are simulated from the non-central chi-square distribution with *k* degrees of freedom and non-centrality parameter $\lambda_\text{ref}$. Subsequently, an estimates of $\hat{\lambda }_b$ is obtained for each $T_b$. Finally, the 2.5th and 97.5th percentile of all $\hat{\lambda }_b$ is determined and used to calculate a 95 % power confidence interval according to Eq. 9.

#### Bootstrap-based diagnostic

A parametric bootstrap procedure can also provide a diagnostic to evaluate the validity of the assumptions underlying the PPE algorithm. The procedure is almost identical to the one described in the previous paragraph, but instead of calculating the power for all $\hat{\lambda }_b$ estimates in the 95 % confidence interval, these estimates are used to plot the cumulative distribution function of the corresponding non-central chi-square distributions. The resulting 95 % confidence band is overlayed with the empirical cumulative distribution function (ECDF) of the test statistics in *T*.

### Algorithm evaluation

The PPE algorithm and its extensions (bootstrap procedure and diagnostic) were evaluated in a simulation study with different pharmacometric models. The evaluation was performed by comparing the performance of the PPE algorithm to the MCPE algorithm for power estimation at a fixed study size (“Bias and precision of MCPE and PPE algorithm” section) as well as in regards to the generation of power curves (“PPE algorithm-based power curves” section). Additionally, the performance of the bootstrap procedure was evaluated regarding its ability to correctly estimate the Monte Carlo uncertainty in the PPE power estimates (“PPE bootstrap procedure” section). Finally, the sensitivity of the diagnostic with respect to the violation of assumptions was tested (“PPE diagnostic” section). All evaluations were performed with a confidence level of 95 %.

#### Evaluation models

The evaluation of the power estimation algorithms was performed based on a simulation study with five different pharmacometric models for different response types: (1) binary, (2) time-to-event (TTE), (3) count, (4) pharmacokinetic (PK) and (5) pharmacokinetic/pharmacodynamic (PKPD). For each model the hypothesis test was performed for a covariate effect of either a dichotomous covariate $z_i$ (binary, TTE and PKPD model) or a continuous covariate $\tilde{z}_i$ (count and PK model). The model equations as well as the parameter values and effect sizes used for this comparison are given in Table 1.

**Table 1:** Models, parameter values ($\theta , \Omega , \sigma$) and effect sizes ($\theta_\text{H}^*$) used for the evaluation of the algorithms

Structural model			Parameter model	Parameter values			
				$\theta_\text{H}^*$	$\theta$	$\Omega$	$\Sigma$
1. Binary							
$$P(y_{ij}|\eta_i)= {\left\{ \begin{array}{ll} p(t_j) & \text{ if } y_{ij}=1 \\ 1-p(t_j) & \text{ if } y_{ij}=0 \end{array}\right. } $$		$p(t)={\text{expit}}{(b + a t)}$	$$\begin{aligned} b &= \theta_{1}+\eta_{i1} \\ a & = (\theta_{2}+\eta_{i2})(1-\theta_\text{H} z_i) \end{aligned}$$	0.3	$$\left( \begin{array}{cc}-1\\ 4\end{array}\right) $$	$$\left( \begin{array}{cc} 0.4 & 0 \\ 0 & 4 \end{array} \right) $$	–
2. Time-to-event (TTE)							
$$\displaystyle P(y_{i})= {\left\{ \begin{array}{ll} h(y_{i})S(y_i) & \text{ if } y_{i}<T \\ S(T) & \text{ if } y_{i}=T \end{array}\right. } $$		$$\begin{aligned}{lcl} h(t) &= \lambda \gamma (\lambda \cdot t)^{\gamma -1}\\ S(t) &= \exp (-\int_0^{t} h(x) dx) \end{aligned}$$	$$\begin{aligned} \lambda & = \theta_{1}\exp (\theta_\text{H} z_i) \\ \gamma & = \theta_{2} \end{aligned}$$	0.4	$$\left( \begin{array}{l}0.2 \\ 2\end{array}\right) $$	–	–
3. Count							
$\displaystyle P(y_{ij}|\eta_i)= \frac{\lambda (t_{j})^{y_{ij}}}{y_{ij}!}e^{-\lambda (t_{j})}$		$\lambda (t) = b + A (1 - \exp (-k\cdot t))$	$$\begin{aligned} b_i &= \theta_{1}\exp (\eta_{i1}) \\ A_i &= \theta_{2}\exp (\eta_{i2}) \\ k_i &= \theta_{3}\exp (\eta_{i3}+\theta_\text{H} \tilde{z}_i) \\ \end{aligned}$$	0.3	$$\left( \begin{array}{ll}1\\ 4\\ 2\end{array}\right) $$	$$\left( \begin{array}{lll} 0.09 & 0 & 0 \\ 0 & 0.09 & 0 \\ 0 & 0 & 0.09 \end{array}\right) $$	–
4. PK [12]							
$\displaystyle y_{ij}=\frac{A_1(t_j)}{V} ( 1 + \varepsilon_{ij})$		$$\begin{array}{lcl} \frac{dA_1}{dt}=\frac{Q}{V_p} A_2 - \left( \frac{Cl}{V} + \frac{Q}{V} \right) {A_1} \\ \frac{d{A_2}}{dt}= \frac{Q}{V} {A_1} - \frac{Q}{V_p} {A_2} \end{array}$$	$$\begin{aligned} Cl &= \theta_{1}\exp (\eta_{i1})(1+\theta_\text{H} \tilde{z}_i) \\ V & = \theta_{2}\exp (\eta_{i2}) \\ Q &= \theta_{3}\exp (\eta_{i3}) \\ V_p & = \theta_{4} \end{aligned}$$	0.2	$$\left( \begin{array}{ll}0.04\\ 0.14\\ 3.62\\ 2.9\end{array}\right) $$	$$\left( \begin{array}{lll} 0.09 & 0 & 0.16\\ 0 & 0.04 & 0\\ 0.16 & 0 & 1.23 \end{array}\right) $$	0.06
5. PKPD [8]							
$$ \begin{aligned} y_{ij}^{\prime} & =\frac{A_2(t_j)}{V} ( 1 + \varepsilon_{1,ij}) \\ y_{ij}^{\prime \prime} & =R(t_j) + \varepsilon_{2,ij} \end{aligned}$$		$$\begin{array}{l} \frac{d{A_1}}{dt}=-k_aA_1 \\ \frac{d{A_2}}{dt}= k_aA_1 - \frac{Cl}{V} A_2 \\ \frac{d{R}}{dt}=k_o\frac{A_2}{V}/(C_{50}+\frac{A_2}{V}) - k_o R \end{array}$$	$$\begin{aligned} Cl & = \theta_{1}\exp (\eta_{i1}) \\ V & = \theta_{2}\exp (\eta_{i2}) \\ k_a & = \theta_3 \\ k_o & = \theta_{4}\exp (\eta_{i3}) \\ C_{50} & = \theta_{5}\exp (\eta_{i4})(1+\theta_\text{H} z_i) \end{aligned}$$	0.3	$$\left( \begin{array}{l} 10\\ 100\\ 2\\ 0.2 \\ 0.3 \end{array}\right) $$	$$\left( \begin{array}{llll} 0.49 & 0 & 0 & 0\\ 0 & 0.49 & 0 & 0\\ 0 & 0 & 0.49 & 0 \\ 0 & 0 & 0 & 0.49 \end{array}\right) $$	$$\left( \begin{array}{l}0.04\\ 0.04\end{array}\right) $$

The study design used for the different models in the simulation study are given in Table 2. For the models with a dichotomous covariate, it was assumed that half the subjects in the study had a covariate value of 0 and the other half a value of 1 (e.g., placebo and treatment group). For the models with a continuous covariate, a normal distribution with mean 0 and standard deviation 1 was assumed for the covariate. The study size $N^*$ used for the evaluation was selected to target roughly 80 % power.

**Table 2:** Study design specifications (study size $N^*$, number and time of observations and dose) used for the algorithm comparison

Model	$N^*$	Observations	Dose
Binary	110	20 equally spaced between 0 and 1	–
TTE	200	1 between 0 and $T=10$	–
Count	160	10 equally spaced between 0 and 1	–
PK	20	9 at 1, 2, 4, 8, 24, 48, 168, 336, 503	150
PKPD	50	3 PK at 0.1, 4, 12 and 3 PD at 4, 6, 12	80

#### Bias and precision of MCPE and PPE algorithm

For all five evaluation models the MCPE and the PPE algorithm were run $L=1000$ times with study size $N^*$ (as specified in Table 2) using 100, 200 and 400 Monte Carlo replicates ($S_M$ in Algorithm 1 and $S_P$ in Algorithm 2). Furthermore, a reference power value $\pi_\text{ref}$ was obtained for each model by running the MCPE algorithm with $S_M=$10,000 replicates.

Measures of bias (relative bias) and precision (standard deviation (SD) and range) were used to summarize the algorithm performance for each model and Monte Carlo sample size. The relative bias was calculated as

12 $$\begin{aligned} \text{bias}\,({\hat{\pi}}_x) = 100 \times \frac{\bar{\pi }_x - \pi_\text{ref}}{\pi_\text{ref}} \end{aligned}$$

the SD as

13 $$\begin{aligned} \text{sd}\,({\hat{\pi}}_x) = \sqrt{\frac{1}{L-1}\sum_{l=1}^{L} ({\hat{\pi}}_{x,l}-\bar{\pi }_x)^2} \end{aligned}$$

and the range as

14 $$\begin{aligned} \text{range}\,({\hat{\pi}}_x) = \max_l {\hat{\pi}}_{x,l} - \min_l {\hat{\pi}}_{x,l} \end{aligned}$$

where $\pi_\text{ref}$ is the reference power, ${\hat{\pi}}_{x,l}$ the power estimate obtained with algorithm *x* ($x \in \{M,P\}$) and $\bar{\pi }_x$ the arithmetic mean of the power estimates $\left( \bar{\pi }_x = \frac{1}{L}\sum_{l=1}^{L}{\hat{\pi}}_{x,l}\right)$.

#### PPE algorithm-based power curves

The ability of the PPE algorithm to obtain full power versus study size curves was evaluated by generating 1000 power curves for all five models based on $S_\text{P}=400$ Monte Carlo samples of study size $N^*$. The median PPE-based power curves were compared to reference power values obtained using the MCPE algorithm with 10,000 replicates at 25, 50, 75, 100 and 125 % of study size $N^*$ (study sizes were rounded to the next even integer value). This comparison was performed graphically.

#### PPE bootstrap procedure

The bootstrap procedure (“Bootstrap procedure to evaluate Monte Carlo uncertainty” section) was evaluated for its ability to characterize the uncertainty due to Monte Carlo noise in the PPE power estimates. For this evaluation the coverage of the bootstrap-based 95 % confidence intervals with 1000 samples was studies for each of the five evaluation models at study sizes $N^*$ using either 100, 200 or 400 Monte Carlo samples for the PPE algorithm. For each model and Monte Carlo sample size, coverage was calculated as the fraction bootstrap-based confidence intervals out of 1000 repetitions containing the reference power value $\pi_\text{ref}$ (determined as specified in “Bias and precision of MCPE and PPE algorithm” section).

#### PPE diagnostic

A formal validation of the bootstrap-based diagnostic procedure (“Bootstrap-based diagnostic” section) is beyond the scope of this manuscript. However, a quick evaluation of its diagnostic power was performed by running the procedure for a scenario representing a violation of the underlying theoretical assumptions.

For this investigation, the binary example from above was modified by using $\theta ^*_H=0.1 instead of 0.3 as before$^1^ and estimating the full model with the constraint $0\le \theta ^*_H$. This way the null-hypothesis is on the boundary of the parameter space and the assumption of a non-central chi-square distribution of the LLR test statistic might not hold. For reference, the diagnostic was also generated without this assumption violation, i.e. $-\infty <\theta ^*_H<\infty$.

### Application example

To illustrate its practical use, the PPE algorithm was implemented using the R plot template functionality of the stochastic simulation and estimation (SSE) tool in PsN version 4.0 and applied to hypothetical example of a phase II Alzheimer’s disease trial evaluating the relative merits of a 12, 18 or 24 months long trial.

The disease progression model was taken from the work of Ito et al. [4, 5] and described the observed disease status for individual *i* at time $t_j$ through the equation

15 $$\begin{aligned} y_{ij}=dp(t_{j}) + pbo(t_{j}) + \epsilon_{ij} \end{aligned}$$

where *dp*() and *pbo*() indicate the disease progression and placebo components described as

16 $$\begin{aligned} dp(t)=S_{0}+\alpha t \end{aligned}$$

and

17 $$\begin{aligned} pbo(t)=A(e^{-k_\text{off}t}-e^{-k_\text{on}t}) \end{aligned}$$

where $S_{0}$ is the baseline disease status and $\alpha$ the disease progression rate. In the placebo response model, *A* is the placebo amplitude and $k_\text{on}$, $k_\text{off}$ are the rate constants for the placebo onset and offset, respectively. The parameters were modeled as follows

$$\begin{aligned} S_{0}&=\theta_1+\eta_{1i} \\ \alpha&=(\theta_2+\eta_{2i})(1-\theta_\text{H}z_i) \\ A&=\theta_3 \\ k_\text{off}&=\theta_4 \\ k_\text{on}&=\theta_5 \end{aligned}$$

where $z_i$ is an indicator variable with 0 in the placebo group and 1 in the treatment group. The parameter values $\theta =(56.4,4.83,-20,2.77,1.73)^T$, $\theta_\text{H}^*=0.3$, $\omega_1^2=14.3$, $\omega_2^2=6.1$, $\omega_{1,2}=-1.2$ and $\sigma ^2=7.9$ were used. These values were in part taken from the publication and partly chosen arbitrarily [4, 5].

A balanced two arm design with placebo and active treatment group was assumed for this example. Visits were scheduled every 6 months for a total study duration of either 12, 18 or 24 months.

### Software

The simulations and estimations for all models in the algorithm comparison were performed in NONMEM 7.3 [1] with the help of PsN version 4.0 [9]. The statistical software R version 3.0.2 [10] was used to implement the PPE algorithm, the source code is given in appendix.

## Results

### Evaluation

#### Bias and precision of MCPE and PPE algorithm

Table 3 compares the relative bias of the MCPE and the PPE-based power at Monte Carlo sample sizes of 100, 200 and 400 for all five evaluation models. Unsurprisingly, as also used when calculating the reference, the MCPE algorithm displayed no major bias in the power calculation [$\text{bias}(\pi_M)<\!\!0.2\,\%$] at any sample size for any of the five models investigated. The bias for the power calculated using the PPE algorithm, was slightly larger and differed between models, but remained small for all models and Monte Carlo sample sizes. The maximal bias of 1.1 % was observed for the PK model. With the exception of the TTE model, the bias for the PPE method was always positive. Furthermore, bias tended to increase slightly with an increasing Monte Carlo sample size.

**Table 3:** Relative bias (%) of power estimates from the Monte Carlo power estimation (MCPE) and parametric power estimation (PPE) algorithm for Monte Carlo sample sizes of 100, 200 and 400

	100		200		400	
	MCPE	PPE	MCPE	PPE	MCPE	PPE
Binary	−0.2	−0.2	0.0	0.1	−0.1	0.1
TTE	0.0	−0.8	−0.1	−0.8	0.0	−0.7
Count	−0.1	0.4	−0.0	0.5	−0.1	0.5
PK	−0.0	1.0	−0.1	1.0	0.0	1.1
PKPD	0.1	0.7	0.1	0.9	−0.0	0.8

The precision of the two algorithms is compared in Table 4. For both algorithms, precision is increasing with an increasing Monte Carlo sample size. At the same Monte Carlo sample size, however, the power estimates obtained using the PPE algorithm were considerably more precise than the MCPE-based estimates. Judging based on the SD, the PPE algorithm required roughly half the number of Monte Carlo samples to achieve the same precision. This finding applied across models and for all samples sizes investigated.

**Table 4:** Precision, in terms of standard deviation (SD) and range, of power estimates from the Monte Carlo power estimation (MCPE) and parametric power estimation (PPE) algorithm for Monte Carlo sample sizes 100, 200 and 400

	SD						Range					
	100		200		400		100		200		400	
	MCPE	PPE	MCPE	PPE	MCPE	PPE	MCPE	PPE	MCPE	PPE	MCPE	PPE
Binary	4.2	2.9	2.9	2.1	2.1	1.5	28.0	18.0	17.5	13.5	15.0	9.3
TTE	3.9	2.5	2.7	1.8	1.9	1.3	28.0	17.5	16.5	11.4	11.8	8.3
Count	4.3	3.1	3.0	2.2	2.1	1.6	28.0	19.9	19.0	13.2	14.3	10.4
PK	4.1	2.9	2.8	2.0	2.1	1.5	25.0	16.8	17.5	11.9	13.2	10.4
PKPD	3.7	2.3	2.6	1.6	1.8	1.1	25.0	14.7	16.5	12.0	11.0	7.2

#### PPE algorithm-based power curves

**Fig. 1:**
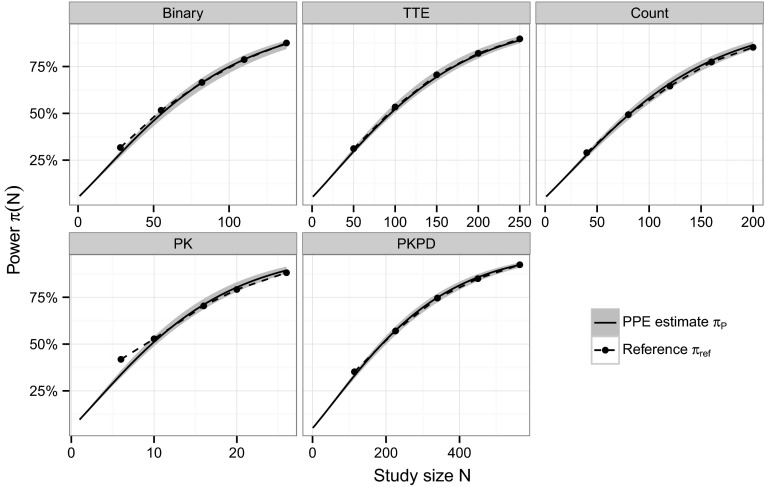
Power versus sample size curves from the parametric power estimation (PPE) algorithm in comparison with the reference power. The *solid black line* indicates the median and the *gray band* represents the 95 % confidence band of the PPE-based power from 1000 runs of the algorithm using 400 Monte Carlo samples. The reference power is indicated by *black dots*

A comparison of power versus sample size curves as obtained with the PPE algorithm and the reference power for all five models is exhibited in Fig. 1. The figure shows the median PPE-based power curve from 1000 repetitions as well as the 95 % confidence band together with the reference. The agreement between reference and median PPE-based power is high across the whole power curve and for all models. Only for the binary and the PK model at the two smallest reference study sizes ($N \le 60$ subjects for binary and $N \le 10$ subjects for PK) a larger deviation is observed. The largest deviation with 8 % was observed for the power estimated using the PK model at $N=6$, all other deviation were smaller than 3 %.

#### PPE bootstrap procedure

**Fig. 2:**
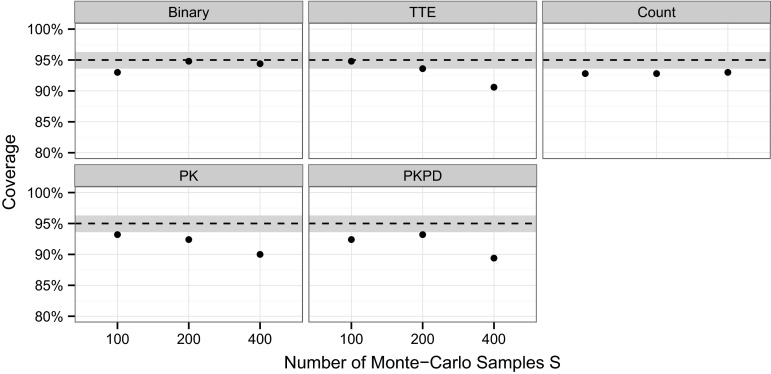
Coverage of the 95 % confidence intervals generated with the parametric power estimation (PPE) bootstrap procedure (shown as *black dots*) for different models and with different Monte Carlo sample sizes. The *dashed lines* indicate the nominal confidence level and the *gray bar* the uncertainty associated with running 1000 repetitions

The results of the coverage evaluation for the PPE bootstrap procedure is shown in Fig. 2. The achieved coverage level for the different models is a reflection of the bias shown in Table 3. For the binary model, with no or minimal bias, the nominal coverage was achieved, while for all other models, with a larger bias in Table 3, the coverage was below the nominal level. The largest deviation from the nominal level was observed for the PKPD model with a coverage 89 %.

Despite these slight deviations from the nominal coverage, the method appears to be sufficiently precise to allow choosing the number of Monte Carlo samples for the PPE algorithm.

#### PPE diagnostic

**Fig. 3:**
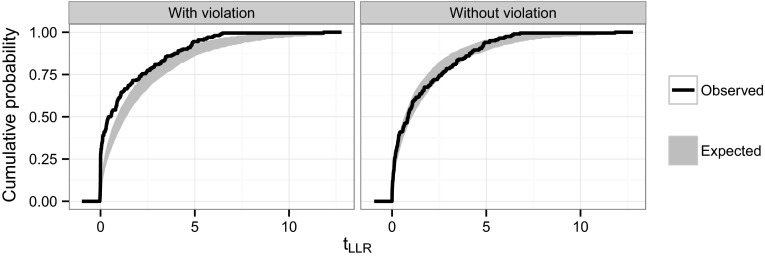
Expected and observed cumulative probability of the log-likelihood test statistic used as a diagnostic for the parametric power estimation (PPE) algorithm. The panels show the diagnostic with and without violation of an assumption underlying the algorithm

Figure 3 shows the bootstrap-based diagnostic when the null hypothesis is forced to be on the boundary of the parameter space and without this restriction. The former violates one of the assumptions required to derive the asymptotic distribution of the test statistic and hence the basis of the PPE algorithm. The diagnostic clearly indicates this violation, showing the ECDF of the test statistic outside the expected confidence band. In the second panel of Fig. 3, where the violation is removed, the ECDF of the test statistic remains within the confidence band.

### Application example

For the preparation of the power study, first, the full and reduced version of the disease progression model described in “Application example” section were implemented in NONMEM and saved as dp24m.mod and dp24m_red.mod, respectively (the r-plots script in PsN uses the convention of a “_red” suffix in the filename to identify the reduced model). Second, a dataset with 100 subjects, two groups (treatment and placebo) and observations at 0, 6, 12, 18 and 24 months was generated in R. Third, the 18 (dp18m.mod and dp18m_red.mod) and 12 months (dp12m.mod and dp12m_red.mod) full and reduced models were created by adding an appropriate IGNORE statement to the 24 months version of the model, e.g. 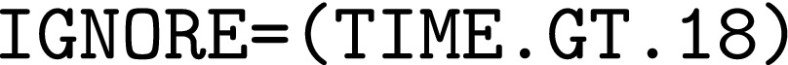 Finally, the necessary steps of data simulation, estimation with all full and reduced models, running of the PPE algorithm and plotting were invoked with the PsN command: 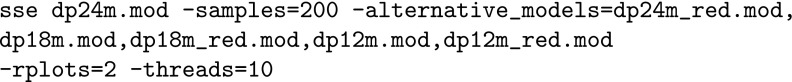 where the -samples=200 argument instructs the software to run 200 Monte Carlo samples with 10 parallel runs (-threads=10). With the -rplots=2 argument, both power versus study size curves and diagnostic curves are produced (-rplots=1 would generate the power curves only).

**Fig. 4:**
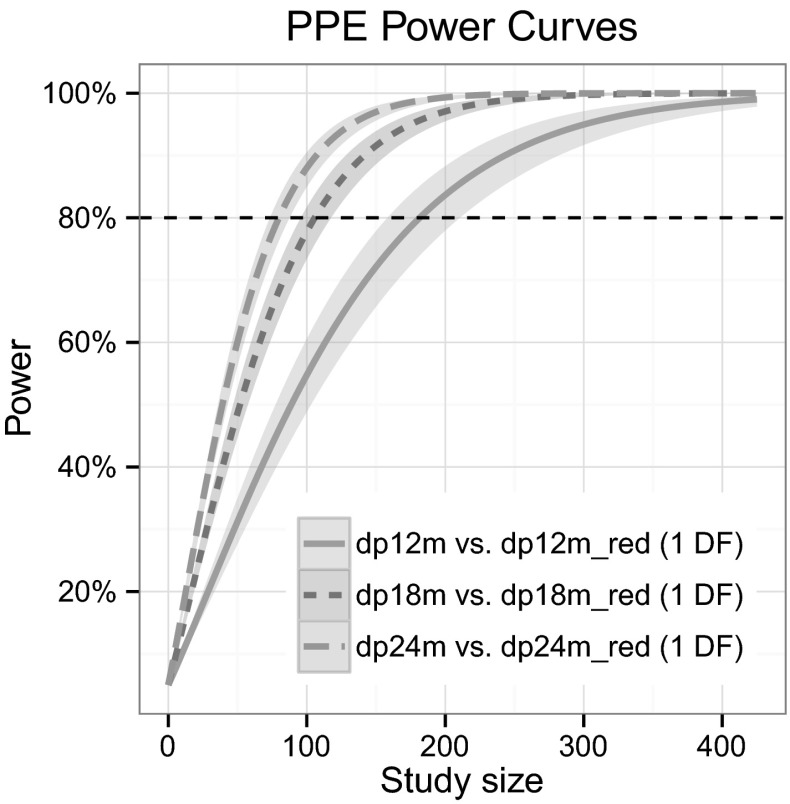
Parametric power estimation (PPE) algorithm-based power versus sample size curves for different study lengths of an Alzheimer’s disease trial automatically generated by the PsN SSE script

The resulting power versus study size graph is shown in Fig. 4, it provides an efficient comparison of the influence of study size and duration on the power to detect a treatment effect. On the cluster system at hand, the full process took about 6 min. As a comparison, power curves generated with the MCPE algorithm using 8 different study sizes per curve would require about 96 min or 16 times longer (8 points per curve and 2 times the number of samples to reach the same precision).

## Discussion

In this work we proposed and evaluated a novel algorithm to estimate the power of a future study. The algorithm estimates the unknown parameter in the theoretical distribution of the test statistic under the alternative hypothesis to obtain more precise estimates with fewer Monte Carlo samples. At a fixed study size, the PPE algorithm required about half as many simulations and estimations to achieve the same level of precision in the power estimate as a purely Monte Carlo-based method. Most importantly, the full power versus study size curve could be obtained from a set of simulations and estimations at a single study size. In addition to that, two routines of practical utility were presented allowing uncertainty evaluation due to Monte Carlo noise as well as an evaluation of the underlying assumptions of the algorithm.

The PPE algorithm derives its advantages from additional assumptions, namely the chi-square distribution and non-central chi-square distribution of the test statistic under the null and alternative hypothesis as well as the proportionality of the non-centrality parameter over the whole study size range. A violation of these assumptions will, therefore, result in a false power prediction. Potential reasons for violations of the two distributional assumptions include pathological hypotheses (as studied for the evaluation of the diagnostic), biased estimators, local minima in the likelihood surface, model misspecifications and numerical problems [15]. The performance of the PPE algorithm is therefore expected to be model, study design and even estimation algorithm dependent. Better performance is generally expected for simple models with rich designs and unbiased, exact-likelihood estimation algorithms. For the models evaluated in this work none of those factors appeared to be a major problem, nevertheless small violations might be the cause for the slight bias observed for all examples. The third assumption of a proportionality of the non-centrality parameter might be violated when extrapolating to or from very small study sizes. This is a probable explanation for the discrepancy between PPE algorithm and reference power for the PK model at a study size of 6. Another possible factor is an increased type-I error for the reference power.

When discussing the bias and discrepancy found for the PPE algorithm, it is important to note that the magnitude observed here ($<$2  %) is of little practical relevance. Generally, the effect of model and parameter uncertainty will be of much larger magnitude than the bias introduced through the additional assumptions of the PPE algorithm. It is also important to acknowledge that the classical MCPE algorithm implicitly relies on the same distributional assumptions when the test statistic is compared to the cut-off from the $\chi ^2$ distribution ($\chi ^2_{1-\alpha , k}$). However, while for the MCPE this assumption can be removed by determining the distribution under the null hypothesis (type I error correction), this might not work for the PPE algorithm. Even if the algorithm can be easily adapted to use a different cutoff value for the hypothesis test, it appears unlikely for the alternative hypothesis to follow the theoretical non-central chi-square distribution when the null hypothesis did not, but this remains to be investigated.

This investigation focuses on simple, uni-variate hypotheses involving fixed effect parameters only, the PPE algorithm, however, extends also to more complex cases. For multivariate, linear hypotheses, for example, it is sufficient to increase the number of degrees of freedom for the chi-square distributions (central and non-central) correspondingly. Hypotheses involving variances of random effect parameters contain some potential theoretical complexities. However, in many practically relevant problems these do not apply and the PPE algorithm should work without problems.^2^ We evaluated this by studying the relative bias of the PPE algorithm for the Count example with an additional random effect on the treatment parameter, i.e. $k_i = \theta_{3}\exp (\eta_{i3}+(\theta_\text{H} + \eta_\text{H}) \tilde{z}_i)$ in Table 1. This scenario corresponds to hypothesis test with one fixed effect parameter and one random effect variance ($\text{H}_0: \theta_\text{H}=0 \wedge \omega_\text{H}^2=0$). The PPE diagnostic did not show any violation and the relative bias was with 0.1, 0.2 and 0.3 % (obtained with 100, 200 and 400 Monte Carlo samples, respectively) similar to the relative bias of the uni-variate case. Nonetheless, it is advisable to judge the results of a power estimation with a complex hypothesis carefully.

This paper also proposes and evaluates the performance of two bootstrap-based procedures, one to judge the influence of the Monte Carlo sample size and one for assumption checking. The former was evaluated by studying the coverage of the method for the five different evaluation models at different Monte Carlo sample sizes. In this evaluation, the procedure did not always show the nominal coverage with deviations of up to 6 %. Results should, thus, be interpreted with caution and resulting confidence intervals be regarded as approximate. Nevertheless, the uncertainty information provides a valuable addition from a practical perspective allowing a quick evaluation whether more Monte Carlo samples are required. The procedure for assumption checking was not formally evaluated. For the example with the null hypothesis on the boundary, the procedure clearly indicated a violation. However, when applied to the other structural models of the paper (results not shown) the diagnostic appeared to be overly sensitive, indicating slight violations in cases where the PPE algorithm performed satisfactorily. An improvement of the diagnostic procedure is therefore a potential focus for future work.

Monte Carlo mapped power (MCMP) as described in the introduction represents an alternative method to obtain power versus study size curves quickly. The runtime comparison of MCMP and PPE is not simple, both algorithms are dependent on a number of settings balancing algorithm speed and precision of the power estimates. A quick evaluation of the time to generate a power curve for the binary model resulted in a average time of 15 m 34 s for the MCMP algorithm and and average time of 23 m 38 s for the PPE algorithm (without parallelization). This comparison was performed based on the results presented by Vong et al. [14] with settings chosen to match the precision achieved with a 200 sample PPE estimate. In practice, the choice of different settings or the parallelization of computations can change these results in either direction. The results are also believed to be model-dependent. A conclusive comparison of both methods’ runtime should therefore be the focus of a future study. However, it seems reasonable to assume that both algorithms have runtimes with the same order of magnitude. The post-processing time, i.e. the sampling for MCMP and the non-centrality parameter estimation for the PPE, is significantly faster for the PPE algorithm. The PPE algorithm is also more transparent about potential violations of the underlying assumptions, as described in the previous paragraph, provides uncertainty information and does not require any special inflated data set. Other advantages of the PPE algorithm are smooth power curves and its gradual operation where results are available with the very first test statistic and then continue to improve. The latter allows users to stop the procedure when a sufficiently precise estimates have been obtained (not yet implemented in PsN) or to add samples and increase precision of an earlier run. Finally, it should be mentioned that both algorithms could be combined, i.e. one could use MCMP to obtain a few samples of the test statistic for one study size and then use the PPE to obtain the full power curve.

Fisher information matrix-based power calculation (FIM-PC) is clearly the fastest method to obtain power curve estimates. However, is a purely asymptotic, does not take the behavior of the estimation algorithm into account and relies on approximations of the Fisher information matrix. The calculation of the expected Fisher information matrix generally requires the implementation of the model in another software and is challenging for categorical data NLMEM. Also, the method does not work if the estimation model is different from the simulation model, such as when a simpler model is to be used for the analysis of the data.

For the future, a formal comparison between PPE, MCMP and FIM-PC would be of value. Furthermore, the PPE algorithm could be extended to be more robust regarding outliers (i.e. through non-successful runs), support sampling-based estimation algorithms (e.g., importance sampling, SAEM) that might lead to negative test statistics or allow for simulating with parameter uncertainty.

## Conclusions

PPE as a novel algorithm to obtain full power versus sample size curves was presented and evaluated. The algorithm is in good agreement with the classical MCPE algorithm and drastically accelerates the generation of full power versus sample size curves for NLMEM.

## Footnotes

1 The violation is more apparent if the alternative hypothesis is close to the boundary

2 When a hypothesis tests the presence of a random effect, i.e. essentially $\text{H}_0: \omega ^2=0$, the null-hypothesis is on the boundary of the parameter space (as variances can not be negative) and one of the assumptions used to derive the theoretical distribution of the test statistic is violated. However, this violation will have minor impact if the power is studied for a parameter value $\theta_\text{H}^*$ that is not too close to the boundary.
